# Experimental simulation of H_2_ coinjection via a high-pressure reactor with natural gas in a low-salinity deep aquifer used for current underground gas storage

**DOI:** 10.3389/fmicb.2024.1439866

**Published:** 2024-07-31

**Authors:** Jean Mura, Magali Ranchou-Peyruse, Marion Guignard, Marion Ducousso, Marie Larregieu, Marie-Pierre Isaure, Isabelle Le Hécho, Guilhem Hoareau, Marie Poulain, Mateus de Souza Buruti, Pierre Chiquet, Guilhem Caumette, Anélia Petit, Pierre Cézac, Anthony Ranchou-Peyruse

**Affiliations:** ^1^LaTEP, E2S UPPA, Université de Pau et des Pays de l’Adour, Pau, France; ^2^IPREM, CNRS, E2S UPPA, Université de Pau et des Pays de l’Adour, Pau, France; ^3^Joint Laboratory SEnGA, E2S UPPA, Pau, France; ^4^LFCR, CNRS, E2S UPPA, Université de Pau et des Pays de l’Adour, Pau, France; ^5^Geosciences Department, Teréga, Pau, France; ^6^Environment Department, Teréga, Pau, France; ^7^Geosciences Department, Storengy, Bois-Colombes, France

**Keywords:** dihydrogen, UGS, deep aquifer, microbial community, gas storage, methanogens, sulfate reducers

## Abstract

If dihydrogen (H_2_) becomes a major part of the energy mix, massive storage in underground gas storage (UGS), such as in deep aquifers, will be needed. The development of H_2_ requires a growing share of H_2_ in natural gas (and its current infrastructure), which is expected to reach approximately 2% in Europe. The impact of H_2_ in aquifers is uncertain, mainly because its behavior is site dependent. The main concern is the consequences of its consumption by autochthonous microorganisms, which, in addition to energy loss, could lead to reservoir souring and alter the petrological properties of the aquifer. In this work, the coinjection of 2% H_2_ in a natural gas blend in a low-salinity deep aquifer was simulated in a three-phase (aquifer rock, formation water, and natural gas/H_2_ mix) high-pressure reactor for 3 months with autochthonous microorganisms using a protocol described in a previous study. This protocol was improved by the addition of protocol coupling experimental measures and modeling to calculate the pH and redox potential of the reactor. Modeling was performed to better analyze the experimental data. As in previous experiments, sulfate reduction was the first reaction to occur, and sulfate was quickly consumed. Then, formate production, acetogenesis, and methanogenesis occurred. Overall, H_2_ consumption was mainly caused by methanogenesis. Contrary to previous experiments simulating H_2_ injection in aquifers of higher salinity using the same protocol, microbial H_2_ consumption remained limited, probably because of nutrient depletion. Although calcite dissolution and iron sulfide mineral precipitation likely occurred, no notable evolution of the rock phase was observed after the experiment. Overall, our results suggested that H_2_ can be stable in this aquifer after an initial loss. More generally, aquifers with low salinity and especially low electron acceptor availability should be favored for H_2_ costorage with natural gas.

## 1 Introduction

Shifting toward carbon-free renewable fuels is essential for facing global warming and resource depletion. Dihydrogen (H_2_) could be a coherent alternative to fossil fuels, as it can be produced by electrolyzers using renewable electricity and does not produce greenhouse gases during its combustion. Thus, excess renewable energy could be used to produce H_2_ (power-to-gas), which could be stored until use in periods of high demand (Pastore et al., 2022). To achieve carbon neutrality, substantial storage capacity will be needed. In France, most seasonal gas storage is assured by underground gas storage (UGS) in deep aquifers, which could provide a large storage capacity for H_2_ in large-scale production. The development of H_2_ production implies that blends of H_2_ and natural gas will flow in the gas grid and will need to be stored in deep aquifers. The H_2_ percentage in these blends is expected to reach 2%. Feasibility and site selection studies of H_2_ storage in porous rock reservoirs and aquifers have been conducted based on the structural characteristics of H_2_ storage, such as porosity, cap rock, and permeability (Aghaei et al., 2023; Bo et al., 2023; Buscheck et al., 2023; Harati et al., 2023; Lysyy et al., 2023; Wang et al., 2023). Many researchers have investigated the injection/withdrawal cycles of H_2_ in aquifers (Heinemann et al., 2021; Chai et al., 2023; Pan et al., 2023; Jadhawar and Saeed, 2023a,b; Izadi Amiri et al., 2024; Saeed and Jadhawar, 2024), enabling the determination of optimal strategies for site exploitation. One of the major challenges of massive H_2_ storage in deep aquifers is its interaction with indigenous microorganisms (Šmigáň et al., 1990; Dopffel et al., 2021; Haddad et al., 2022b; Mura et al., 2024). Indeed, H_2_ is a reactive molecule that can be used as an energy source and an electron donor by autochthonous lithoautotrophic microorganisms, so called hydrogenotrophs (Šmigáň et al., 1990; Dopffel et al., 2021; Haddad et al., 2022b; Mura et al., 2024). Based on town gas storage experience (Šmigáň et al., 1990; Buzek et al., 1994; Liebscher et al., 2016; Stolten and Emonts, 2016; Pichler, 2019; Tremosa et al., 2023) and experimental studies (Haddad et al., 2022b; Dohrmann and Krüger, 2023; Mura et al., 2024; Vasile et al., 2024), the major reactions to be expected are sulfate reduction, methanogenesis (Vítězová et al., 2023), and acetogenesis with acetate and formate production. The electron acceptors of these reactions (sulfate, CO_2_, and bicarbonates; Ebrahimiyekta, 2017) can be naturally present at various concentrations in deep aquifers and can be found in aquifers hosting UGS storages. In addition to energy loss through H_2_ consumption, microbial reactions and growth can cause multiple drawbacks to H_2_ storage. Biofilm development can lead to pore clogging (Eddaoui et al., 2021) and has been shown to influence sandstone wettability and liquid/gas transfer (Stewart, 2003; Ali et al., 2023). Sulfate reduction produces sulfide and can lead to souring. Physicochemical changes induced by microbial reactions could lead to precipitation and dissolution by equilibrium displacement (Ulrich et al., 2003; Dupraz et al., 2009) or lasting changes in water physicochemistry (i.e., low sulfate concentration; Ranchou-Peyruse et al., 2019). An induced decrease in porosity would lower the storage performance, whereas an increase in porosity could harm the storage integrity. However, microbial activity can be inhibited by inappropriate pH or a lack of nutrients (Dopffel et al., 2023; Mura et al., 2024). The abiotic effects of H_2_ could also be expected, as H_2_S could be produced by pyrite reduction (Šmigáň et al., 1990). However, microbial activity could also benefit storage, as microorganisms such as sulfate reducers are known to attenuate aromatic molecules (Mancini et al., 2003).

Despite the development of new experimental studies (Haddad et al., 2022b; Dohrmann and Krüger, 2023; Dopffel et al., 2023; Liu et al., 2023; Mura et al., 2024; Vasile et al., 2024), the feasibility of H_2_ storage in porous reservoirs remains unclear, mainly because of site-dependent phenomena related notably to its pressure and temperature conditions and its mineralogy. While microorganisms are expected to be present in deep aquifers, their taxonomic diversity and cell concentration could change depending on the studied site. Moreover, there is variability in the physicochemistry and hydrology between aquifers (Fillinger et al., 2023), and temperature and mineralogy can influence microbial activity (Dohrmann and Krüger, 2023; Muller et al., 2023). Thus, site-specific microbiological and geochemical analyses are needed to evaluate the feasibility of H_2_ coinjection in deep aquifers (Dopffel et al., 2021). For experimental approaches, the modeling of H_2_ storage in porous rocks has increased (Amid et al., 2016; Hagemann et al., 2016; Hogeweg et al., 2022; Veshareh et al., 2022; Gelencsér et al., 2023; Maniglio et al., 2023; Strobel et al., 2023; Tremosa et al., 2023; Wu et al., 2023). Conceptual kinetic modeling of microbial growth and reactions was performed on various systems ranging from near atmospheric pressure laboratory experiments to reservoir-scale modeling. Both experimental and modeling approaches underscore the need for additional experimental studies and data to better understand the intricate phenomena of H_2_ in underground storage.

In recent works (Haddad et al., 2022b; Ranchou-Peyruse et al., 2023; Mura et al., 2024), a multidisciplinary protocol to recreate deep aquifers in a three-phase high-pressure reactor was developed and applied to four French aquifers hosting natural gas storages. In this work, a similar approach was applied to another UGS in a deep aquifer to investigate the feasibility of H_2_ coinjection at this site. The formation water and rock phase were sampled from the studied aquifer, and a gas mixture representing the natural gas storage (with 1% CO_2_) was added with a final pressure of 60 bar. Two percent H_2_ was added to the gas phase to simulate its injection in the aquifer. The extent of the reactions was computed based on physicochemical parameters and microbial taxonomic diversity to model the amount of H_2_ consumed by each microbial reaction. To better quantify the *in situ* pH and redox potential in the high-pressure reactor, the initial protocol was upgraded with a new sampling protocol coupled with modeling using PHREEQC. Briefly, dissolved gases were quantified at atmospheric pressure to account for their acido-basic and redox effects. The Materials and Methods section describes the experimental protocol and the modeling approach. The outcomes of the experiment and modeling results are presented in the Results section and analyzed and compared in the Discussion section.

## 2 Materials and methods

### 2.1 Simulated site characteristics and sampling procedure

The UGS studied in this work is a previously studied deep aquifer operated for seasonal natural gas storage, referred to as Ab_L in the literature (Ranchou-Peyruse et al., 2019, 2024; Haddad et al., 2022a, 2023). This aquifer is in southwestern France and belongs to the South Aquitaine sedimentary basin (582 m depth; Haddad et al., 2022a). The formation water was sampled from a control well close to the gas bubble, ensuring optimal contact between the liquid phase (i.e., formation water) and the stored natural gas. With 0.8% salinity of seawater, the formation water has a low salinity (Haddad et al., 2022a). The porous rock is mainly composed of quartz (80%) and calcite (12%) and contains small amounts of iron sulfide, clays, and barite (Jacquemet et al., 2020; Haddad et al., 2022a). During the sampling campaign on well Ab_L_1, formation water was collected using two bottom hole samplers (Leutert Bottom Hole Positive Displacement Sampler) to ensure sterile conditions, maintenance of anoxia and pressure until the samples reached the laboratory, according to a protocol (Ranchou-Peyruse et al., 2023). From the samplers, 578 mL and 594 mL were slowly depressurized (<1 bar.min^−1^) in the laboratory, transferred to sterile and anoxic flasks, and stored at 4°C until use. On the day of sampling, wellhead formation water was also sampled and sterilized by filtration (PES 47 mm membranes, 0.1 μm, Sartorius). All water samples were stored at 4°C in darkness until use. On the day the experiment began, the two flasks were mixed with 778 mL of filtered water collected from the wellhead. The procedure was carried out in an anaerobic chamber (GP Campus, Jacomex). One hundred milliliters were sampled to study the microbial taxonomic diversity. The characteristics and composition of the water mix are detailed in Table 1. The rock phase was composed of infra-molassic sands and was recovered from drilling cuttings from the reservoir studied (Ranchou-Peyruse et al., 2019; Haddad et al., 2022a, 2023).

**Table 1:** Characteristics and composition of the formation water sampled from the studied UGS aquifers.

Experiment parameters	Value	Unit
**Water composition**		
pH	8.3	
Redox potential	−20	mV
Chloride	0.23	mM
Nitrate	<0.0016	mM
Nitrite	<0.0004	mM
Sulfate	0.09	mM
Carbonate	<1	mM
Bicarbonate	3.44	mM
Calcium	1.37	mM
Total iron	9.24	μM
Magnesium	0.30	mM
Potassium	0.15	mM
Sodium	0.50	mM

Analyses were carried out at atmospheric pressure by UT_2_A (Pau, France).

### 2.2 Experiment

#### 2.2.1 Experimental protocol

This experiment was carried out in a high-pressure reactor using a protocol to simulate H_2_ or O_2_ injection in deep aquifers used previously (Haddad et al., 2022a,b, 2023; Mura et al., 2024). This reactor was made of corrosion-resistant Hastelloy C-276 material and was equipped with pressure and temperature sensors. The solid phase was contained in a Teflon basket sitting in the middle of the reactor to represent the water-gas interface in the porous rock of the aquifer. A piston enables the modification of the reactor volume to compensate for the liquid sampling and manage the height of the water-gas interface throughout the experiment. The liquid and gas phases were stirred at 20 rpm. After the solid basket filled with rock was introduced into the reactor, the reactor was sterilized by moist heat with ultrapure water at 110°C for 24 h under low nitrogen (N_2_) pressure. Then, the liquid phase was introduced into the reactor previously placed under vacuum from a Teflon bottle prepared in an anaerobic glovebox. A custom gas mixture simulating natural gas (99% CH_4_, 1% CO_2_, 7.95 ppm benzene, and 3.57 ppm toluene) was injected into the reactor to reach the target pressure. The height of the piston was set to immerse the solid phase fully. After 7 days, the piston was lowered to immerse 1 cm of solid. The pressure loss induced was compensated by injecting a new amount of the initial gas mixture. After validation of the microbial activity, dihydrogen (CAS: 1333-74-0, purity 99.999%) was injected at a gas phase molar fraction of 2%. The microbial activity was validated by sulfate consumption and cell count (data not shown) based on previous experiments (Haddad et al., 2022a,b, 2023; Mura et al., 2024). Immediately after H_2_ injection, additional filtered formation water was injected into the reactor to extend the duration of the experiment, which was limited by the quantity of water available to the sample. Each week, the composition of the liquid and gas phases was monitored, and more detailed chemical and biological analyses (qPCR, taxonomic diversity, and benzene/toluene quantification) were performed for key events. Water samples were taken regularly to monitor changes in the taxonomic and functional diversities of the microbial community. Filtered formation water was added to the reactor on day 17 of the experiment to extend its duration. At the end of the experiment, the remaining liquid phase and the solid basket were maintained under anoxic conditions and recovered for further analysis (Haddad et al., 2022a).

#### 2.2.2 Physico-chemical analyses

The anions (fluoride, acetate, formate, chloride, sulfate) were analyzed by ionic chromatography (Dionex Integrion HPIC, Thermo Fisher Scientific) with ±5% precision. The dissolved inorganic carbon, including carbonate, bicarbonate, and dissolved CO_2_, was quantified using a dedicated chromatography system (ICS-900, Dionex) equipped with an ICE-AS1 IC column. The precision of this measure was ±5%. The metal concentrations (sodium, iron, barium, magnesium, potassium, and calcium) in the liquid samples were determined during all the experiments via inductively coupled plasma-optical emission spectroscopy (ICP–OES, Thermo Scientific iCAP6500 Duo). The instrumental conditions for ICP–OES were as follows: RF power of 1,300 W, plasma gas flow rate of 15 L.min^−1^, nebulizer gas flow rate of 0.65 L.min^−1^, and auxiliary gas flow rate of 0.5 L.min^−1^. All the samples were diluted in a 2% HNO_3_ solution at a dilution ratio of four. The measurements were performed in triplicate, and the coefficient of variation (relative standard deviation) for each analysis was less than 2% for all measurements.

The gas phase species (H_2_, O_2_, CH_4_, CO_2_, H_2_S) were quantified using in-line micro gas phase chromatography with 5% precision (GC-mTCD; Micro GC Fusion; Chemlys; France). The pH and redox potential were measured at atmospheric pressure using Inlab Ultramicro ISM (Mettler Toledo) and Inlab Redox Micro (Mettler Toledo) probes. The specifications of each analysis method were detailed in a previous work (Haddad et al., 2022a).

The use of the compound-specific isotope analysis (CSIA) approach makes it possible to demonstrate or estimate the *in situ* bioattenuation of organic pollutants such as benzene and toluene (Fischer et al., 2016; Ponsin et al., 2017). This method can be used to directly monitor the biodegradation of aromatic hydrocarbons in groundwater by measuring the isotopic fractionation of the remaining contaminant as degradation proceeds (Mancini et al., 2003). CSIA requires an analytical chain composed of a gas chromatograph (GC, Thermo, Trace 1310) coupled via a combustion interface at 1,000°C (CT, Thermo, GC-Isolink) to an isotope ratio mass spectrometer (IRMS, Thermo Delta V Plus). All δ^13^C signatures of the analytes were reported relative to Vienna PeeDee Belemnite (δ^13^CVPDB), and the calibration was achieved by coupling an elemental analyzer (EA, Thermo, Flash 2000) with an isotope ratio mass spectrometer (Berg et al., 2007; Hunkeler et al., 2008). In parallel, the identification and quantification of benzene and toluene in the liquid and gas phases were carried out by coupling gas chromatography to a quadrupole mass spectrometer (MS, Thermo, ISQ). This configuration thus makes it possible to obtain the identification and quantification of benzene/toluene via GC–MS and the determination of isotopic ratios via GC-CT-IRMS during the analysis of a sample, avoiding any problem of correspondence between the CO_2_ peak and the compound of interest. Preconcentration of benzene and toluene was performed by SPME with a polydimethylsiloxane/carboxene (PDMS/CAR) fiber, and chromatographic separation was performed with a DB-624 column (Agilent). For each sample, two 10 mL water samples were taken and stored at 4°*C. prior* to the analysis, 90 μL of the 0.5 ppm 1,2,4-trimethylbenzene internal standard was added to each 6.9 g sample. Gas samples were collected in vials at one bar using a needle sampling system controlled by a manometer. The quantification was performed with methane as a reference gas containing 10 mol ppm benzene and toluene (Li et al., 2014; Haddad et al., 2022a).

#### 2.2.3 Molecular biology approaches

##### 2.2.3.1 Nucleic acid extraction and RNA reverse transcription

Aqueous phase samples were used to coextract nucleic acids (DNA and RNA) throughout the experiment. Membrane filters (47 mm PES with 0.1 μm porosity, Sartorius Stedim) were used to filter aqueous samples directly from the reactor. The filters with the samples were kept at −80°C until use. The filters were then ground in liquid nitrogen, and a Fast RNA Prosoil Direct kit (MP BIO) was used to recover the nucleic acids. An AllPrep RNA/DNA kit (Qiagen) was used to separate the DNA and RNA. A Quant-it™ dsDNA HS kit (Invitrogen) and a Quant-it™ RiboGreen kit (Invitrogen) were used to quantify the extracted DNA and RNA, respectively. A BioTEK SYNERGY HTX microplate reader was used to measure the extracted DNA and RNA. M-MLV reverse transcriptase (Invitrogen™) was used to reverse transcribe RNA and obtain complementary DNAs (cDNAs).

##### 2.2.3.2 Polymerase chain reaction, qPCR, and sequencing

The primer pairs 515F-928R (V4-V5 region), dsr2060F-dsr4R, and mlasF-mcrAR (Wagner et al., 1998; Geets et al., 2006; Steinberg and Regan, 2008, 2009; Wang and Qian, 2009) were used to target the *16S rRNA*, *dsrB* and *mcrA* genes from the obtained DNA and cDNA, respectively. The addition of bovine serum albumin (BSA, NEV-B9200S) to PCR at a concentration of 1 mg.mL^−1^ reduced the inhibition. A Taq PCR kit (Roche) was used to amplify the *16S rRNA* and *dsrB* genes, while a Fidelio® Hot Start PCR kit (Ozyme) was used to amplify the *mcrA* gene. Amplifications were obtained using a 2700 Thermal Cycler (Applied Biosystems). Haddad et al. (2022a) described the procedures in greater detail. The quantification of genes, their transcripts and associated standards was performed by quantitative PCR (qPCR; Bio-Rad CFX Connect) and 41 Takyon NO ROX SYBR 2X MasterMix blue dTTP (Eurogentec), as described by Haddad et al. (2022a). The primer pairs used in this work were synthesized with the adaptors GTGYCAGCMGCCGCGGTA (forward) and CCCCGYCAATTCMTTTRAGT (reverse). The raw sequencing data are publicly accessible on the NCBI SRA with bioproject ID PRJNA1117242. The MiSeq sequencing data were processed with QIIME 2 (Bolyen et al., 2019; version 2022.11) to analyze taxonomic diversity. Amplicon sequence variants (ASVs) were obtained with DADA2 (Callahan et al., 2016) after demultiplexing, filtering, denoising, trimming of any eliminating chimera sequences and excluding singletons. The SILVA v138 database (Quast et al., 2012; Yilmaz et al., 2014) was used for taxonomic affiliation. The same treatment was applied to the *mcrA* and *dsrB* sequences, as well as the *16S rDNA* sequences. For *mcrA*, the Yang et al. (2014) database was used for ASV affiliation, while for *dsrB*, we used our own database (Ranchou-Peyruse et al., 2024). Calculations and analyses in R.Studio (version 4.2.2) were performed with the Phyloseq (McMurdie and Holmes, 2013) and ggplot 2 (Wickham, 2016) packages. The ComplexHeatmap package (Gu et al., 2016; Gu, 2022) was used to generate heatmaps, and the Corrplot (Wei and Simko, 2017), FactoMineR (Lê et al., 2008) and factoExtra (Kassambara and Mundt, 2020) packages were used for PCA and PCoA. Bray–Curtis was used for PCoA distance calculations, and covariance analysis was used for PCA.

#### 2.2.4 X-ray diffraction

X-ray diffraction (XRD) was performed to characterize the crystallized mineralogical phases before and after the experiment. At ambient temperature in the anaerobic glove box, samples were collected from the basket at three different depths (surface 0–1 cm, middle 2–5 cm, and bottom 6–7.5 cm). The samples were then dried with N_2_ gas flux, manually ground, and sieved to <100 μm into a homogeneous powder in the anaerobic chamber to limit oxidation. Solid powders were then mounted on holders and directly analyzed by XRD. The analyses were performed using a Bruker D2 Phaser powder diffractometer equipped with a Cu Kα radiation source. XRD patterns were recorded over a 5° to 90° 2Θ range with a 0.02° step and a 0.5 s counting time per step. DIFFRAC.EVA software was used to identify the mineral phases.

#### 2.2.5 Scanning electron microscopy

Aliquots of the same samples collected at three different depths were subjected to a petrographic study using scanning electron microscopy coupled with energy dispersive spectroscopy (SEM–EDS). Solid pieces were directly mounted on PIN stubs and coated with carbon. Observations and mineral identification were performed with a JEOL JSM 7800F Prime SEM-FEG instrument equipped with an Oxford Instruments AZtecEnergy EDS SDD X-Max 80 mm^2^ detector at Centre Castaing, Toulouse, France.

#### 2.2.6 Biochemical modeling

To better identify microbial reactions and quantify their effect on H_2_ consumption, the extent of reactions was computed. The sulfate reduction (van Houten et al., 1994; Reitenbach et al., 2015; Hemme and Van Berk, 2018; Eq. 1), acetogenesis (Gregory et al., 2019; Rabii et al., 2019; Eq. 2), formate production (Logroño et al., 2022; Eq. 3), and methanogenesis (Panfilov, 2010; Hemme and Van Berk, 2018; Thaysen et al., 2021; Eq. 4) reactions were chosen based on literature mechanisms and used in agreement with microbiology analyses. These reactions were combined with the acido-basic reaction of the $CO_{2}$/$HCO_{3}^{-}$ couple to consider the buffering of H^+^ ion consumption/production. The stoichiometric coefficients of HS^−^ and H_2_S were calculated based on sulfide speciation (Daumas et al., 1993; Mura et al., 2024). The extent of each reaction was determined from the quantification over time of selected species. The extents of sulfate reduction, acetogenesis, formate production, and methanogenesis were calculated based on the experimental changes in the quantities of sulfate, acetate, formate, and CO_2_, respectively.


(1) $$\begin{array}{l} SO_{4}^{2-}+4H_{2}+1.93\;CO_{2}\to 0.93H_{2}S\\ +0.07\;HS^{-}+1.93\;HCO_{3}^{-}+2.07\;H_{2}O \end{array}$$

(2) $$CO_{2}+HCO_{3}^{-}+4H_{2}\to CH_{3}COO^{-}+3H_{2}O$$

(3) $$HCO_{3}^{-}+H_{2}\to HCOO^{-}+H_{2}O$$

(4) $$CO_{2}+4H_{2}\to CH_{4}+2H_{2}O$$

To consider the evolution of dissolved gas, the liquid–gas equilibrium was modeled with PHREEQC version 3 software (Parkhurst and Appelo, 2013) using the phreeqc.dat database. This database uses the Peng Robinson equation of state (Peng and Robinson, 1976) to consider the gas phase’s nonideality. Gas solubility was calculated based on the fugacity of gases in the gas phase and the hypothesis of thermodynamic equilibrium between the two phases. This modeling procedure was detailed in a previous work (Mura et al., 2024).

The standard Gibbs energy of reaction ($\Delta G_{r}^{0})$ of formate production (Eq. 3) was computed using the NBS Tables of Chemical Thermodynamics (Wagman, 1982), considering standard conditions of 298.15 K and 1 bar (Eq. 7). Activities were computed using the PHREEQC data from the *in situ* characterization protocol (section 0.). The Gibbs energy of reaction at the temperature of the experiment [$\Delta G_{r}^{0}\left(T\right)$] (36°C) was computed using the Gibbs-Helmoltz equation (Eq. 8). The theoretical equilibrium concentration of formate in the reactor was obtained by computing the activity of formate at equilibrium with dissolved H_2_ and bicarbonate using the formate production mass action law (Eq. 9). The theoretical equilibrium concentration of formate at atmospheric pressure was calculated considering the activity of dissolved H_2_ in equilibrium with a gas phase containing the molar fraction of H_2_ measured on day 21 at 1 bar.

#### 2.2.7 *In situ* characterization protocol

In this work, a protocol combining experimental measures and modeling was developed to determine the *in situ* pH and redox potential of a reactor. A 10 mL liquid sample was collected from the reactor in a gas-tight glass syringe (SGE, 50 mL) closed by a manual valve to retain the gases that were degassed due to a change in pressure. The total released gas quantity was quantified by directly reading its volume via syringe graduation. The uncertainty of this measure was ±5% compared to a reference value measured by a manual gasometer (results not shown; VINCI Technologies). The sample temperature was measured by a CheckTemp thermometer (± 0.2°C; HANNA instruments). Both the liquid and gas phases were injected into a rubber-sealed vial placed under an N_2_ atmosphere for analysis. The gas composition in the vial was determined using the micro gas chromatography setup described in section 2.2.2. It was assumed that the gas composition did not change between the syringe and the vial, except for the dilution in N_2_. The liquid phase was analyzed using the methods described in section 2.2.2.

The *in situ* conditions were then modeled using PHREEQC with the BRGM database Thermoddem (Blanc et al., 2012). This database was modified by removing redox couples linked by slow redox reactions (carbon, nitrogen, and sulfur) to model redox disequilibrium in solution (Gelencsér et al., 2023; Tremosa et al., 2023). This modeling was composed of three steps. First, the liquid and the released gas at atmospheric pressure were modeled using experimentally collected data. Using the ideal gas law, each gas quantity was computed from the gas composition, pressure, temperature, and volume. It was assumed that the sample in the syringe was at atmospheric pressure. In the second step, the thermodynamic equilibrium between the gas and liquid phases in the syringe was modeled using the composition of the syringe gas phase to quantify the dissolved gas concentrations in the syringe. At this stage, the calculated and measured pH values were close. Finally, the pressurization of the two phases at the reactor pressure and temperature was modeled to return to the reactor conditions.

The pH under these conditions was determined by the default PHREEQC calculations, assuming acido-basic equilibrium among all acid–base species in the solution. In the absence of redox equilibrium in solution, it is possible to only compute the theoretical Nernst potential of each redox couple (Lindberg and Runnells, 1984; Stefánsson et al., 2005; Ioka et al., 2011). Using Thermoddem.dat parameters, the redox potentials of the H^+^/H_2_ and CO_2_/CH_4_ couples were calculated (Eqs. 5, 6):


(5) $$E_{H^{+}/H_{2}}=\left(\log\left(K_{H^{+}/H_{2}}\right)+\log\left(\frac{1}{a_{H_{2}}}\right)-2pH\right)\ln\left(10\right)\frac{RT}{2F}$$

(6) $$E_{CO_{2}/CH_{4}}=\left(\log\left(K_{CO_{2}/CH_{4}}\right)+\log\left(\frac{a_{CO_{2}}}{a_{CH_{4}}a_{H_{2}O}^{2}}\right)-8pH\right)\ln\left(10\right)\frac{RT}{8F}$$

with

$E_{i}$: Redox potential of couple i relative to the standard hydrogen electrode (V).
$\log\left(K_{i}\right)$: Equilibrium constant of the half equation of couple i.
a: activity of species i.
R: gas constant (8.314 kg m^2^ s^−2^ mol^−1^ K^−1^).
T: temperature (K).
F: Faraday constant (9.6485∙10^4^ A s mol^−1^).

(7) $$\Delta G_{r}^{0}=\sum_{i}\nu_{i}\Delta G_{f}^{0}{\;}_{i}$$

(8) $$\Delta G_{r}^{0}\left(T\right)=\frac{\Delta G_{r}^{0}}{T_{ref}}+\Delta H_{r}^{0}\left(\frac{1}{T}-\frac{1}{T_{ref}}\right)$$

(9) $$K\left(T\right)=\frac{a_{HCOO^{-}{\;}_{\left(aq\right)}}a_{H_{2}O_{\left(aq\right)}}}{a_{H_{2}{\;}_{\left(aq\right)}}a_{HCO_{3}^{-}{\;}_{\left(aq\right)}}}=\frac{e^{\Delta G_{r}^{0}\left(T\right)}}{RT}$$

with

$\nu_{i}$: Stoichiometric coefficient of species i in the reaction.
$\Delta G_{f}^{0}{\;}_{i}$: Standard Gibbs energy of formation of species i.
$T_{ref}$: Standard state temperature.
T: Temperature of the experiment.
$\Delta H_{r}^{0}$: Standard enthalpy of reaction.
K(T): Equilibrium constant.
$a_{i}$: Activity of species i.

## 3 Results

### 3.1 Physicochemical monitoring of the gas phase evolution during the experiment

**Figure 1:**
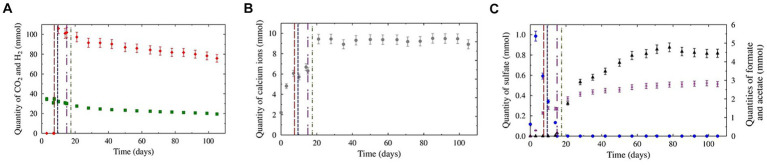
Evolution of the aqueous and gaseous phase compositions during the experiments. The red, blue, purple, and green dotted lines represent CH_4_ + CO_2_ gas reinjection, H_2_ injection, total sulfate consumption, and formation water addition, respectively. Panel **(A)** represents the evolution of H_2_ (red diamonds) and CO_2_ (green squares) in the gas phase. Panel **(B)** represents the evolution of sulfate (blue dots), formate (black triangles), and acetate (purple crosses) in the liquid phase. Panel **(C)** represents the evolution of calcium ions in the liquid phase.

The CH_4_-CO_2_-benzene/toluene gas mixture was first injected to a total pressure of 62.0 ± 0.6 bar at 36°C, corresponding to 4.54 ± 4.5∙10^−2^ moles of CH_4_ (results not shown) and 4.58∙10–2 ± 4.6∙10–4 moles of CO_2_ (Figure 1A). After 7 days, mainly due to solubilization, the CO_2_ quantity decreased to 3.1∙10^−2^ ± 1.5∙10^−3^ mol, while the CH_4_ quantity remained within the error margin due to its low solubility. On day 7, the piston was lowered to immerse only 1 cm of the solid basket. To compensate for the pressure drop caused by the increase in the cell volume, the initial gas was injected again, resulting in a total quantity of 4.7 ± 2.3∙10^−1^ moles of CH_4_ and 3.3∙10^−2^ ± 1.7∙10^−3^ moles of CO_2_. Dihydrogen was injected on day 9, with a molar fraction of 2.2 ± 0.1%, corresponding to 1.06∙10^−1^ ± 5.3∙10^−3^ moles (Figure 1A). On day 17, 353 g of formation water was added to the reactor to prolong the experiment. Following this injection, on day 21, only CO_2_ solubilization was significant (2.7∙10^−2^ ± 1.4∙10^−3^ moles). From day 21 to the end of the experiment (day 105), the H_2_ and CO_2_ concentrations slowly decreased to 7.6∙10^−2^ ± 3.8∙10^−3^ and 1.95∙10^−2^ ± 9.8∙10^−4^ mole, respectively. No significant evolution of CH_4_ was noted.

### 3.2 Physicochemical monitoring of the liquid phase evolution during the experiment

Before injection into the reactor, the formation water contained mostly bicarbonate (2.4∙10^−3^ ± 1.2∙10^−4^ mol.kg^−1^) and calcium (Figure 1B; 1.31∙10^−3^ ± 6.5∙10^−5^ mol.kg^−1^). Sodium (6.0∙10^−4^ ± 3.0∙10^−5^ mol.kg^−1^), magnesium (4.4∙10^−4^ ± 2.2∙10^−5^ mol.kg^−1^), chloride (3.0∙10^−4^ ± 1.5∙10^−5^ mol.kg^−1^), and sulfate (Figure 1C; 7.0∙10^−4^ ± 3.5∙10^−5^ mol.kg^−1^) were also present initially at lower concentrations. Iron and barium were detected at trace levels (6.5∙10^−6^ ± 3.2∙10^−7^ mol.kg^−1^ and 1.0∙10^−6^ ± 5.0∙10^−7^ mol.kg^−1^, respectively). Acetate and formate were not detected. On day 3, after contact between the liquid and solid phases, significant increases in bicarbonate (3.1∙10^−3^ ± 1.5∙10^−4^ mol.kg^−1^), calcium (2.88∙10^−3^ ± 1.4∙10^−4^ mol.kg^−1^), chloride (1.97∙10^−3^ ± 9.84∙10^−5^ mol.kg^−1^) and sulfate (5.9∙10^−4^ ± 2.9∙10^−5^ mol.kg^−1^) concentrations were observed. Acetate was first detected on this day (1.82∙10^−4^ ± 9∙10^−6^ mol.kg^−1^). From days 3 to 9, before H_2_ injection, sulfate molality decreased to 3.7∙10^−4^ ± 1.8∙10^−5^ mol.kg^−1^. Furthermore, the bicarbonate, calcium, and acetate concentrations increased to 4.9∙10^−3^ ± 2.4∙10^−4^ mol.kg^−1^, 4.1∙10^−3^ ± 2.1∙10^−4^ mol.kg^−1^, and 7.0∙10^−4^ ± 3.5∙10^−5^ mol.kg^−1^, respectively. The other ion concentrations remained within the margin of error.

After H_2_ injection, sulfate molality continued to decrease until total consumption on day 15. On day 21, after the addition of the formation water, several ion molalities increased: Bicarbonate (6.6∙10^−3^ ± 3.3∙10^−4^ mol.kg^−1^), calcium (4.8∙10^−3^ ± 2.4∙10^−4^ mol.kg^−1^), acetate (1.08∙10^−3^ ± 5.4∙10^−5^ mol.kg^−1^) and formate (1.12∙10^−3^ ± 5.6∙10^−5^ mol.kg^−1^). After day 21, the bicarbonate and calcium concentrations continued to increase until they reached a plateau on day 28 (7.1∙10^−2^ ± 3.6∙10^−4^ mol.kg^−1^ and 4.9∙10^−3^ ± 2.4∙10^−4^ mol.kg^−1^, respectively). From detection on day 14, formate molality steadily increased to a maximum molality of 3.4∙10^−3^ ± 1.7∙10^−4^ mol.kg^−1^ before slightly decreasing at the end of the experiment (3.1∙10^−3^ ± 1.5∙10^−4^ mol.kg^−1^). Acetate production continued after H_2_ injection but at a diminishing rate. At the end of the experiment, the acetate molality stabilized at 1.74∙10^−3^ ± 8.7∙10^−5^ mol.kg^−1^. The iron and barium concentrations steadily increased during the experiment, reaching 1.83∙10^−4^ ± 9.14∙10^−6^ mol.kg^−1^ and 1.95∙10^−5^ ± 9.7∙10^−7^ mol.kg^−1^, respectively, at the end of the experiment.

### 3.3 Benzene and toluene evolution

**Figure 2:**
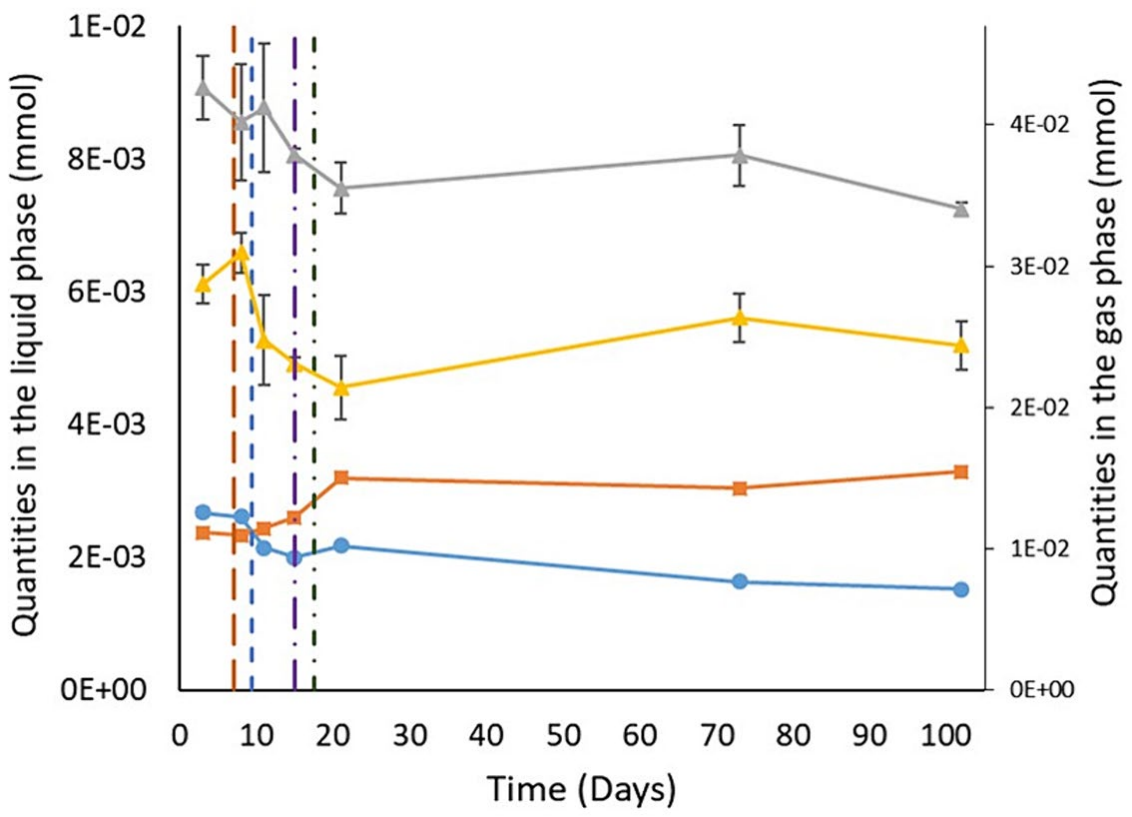
Variations in benzene and toluene quantities in the liquid and gas phases. The gray and yellow curves show the evolution of the quantity of benzene and toluene in the gas phase, respectively. The light blue and orange curves correspond to the quantity of benzene and toluene in the liquid phase, respectively. The brown, blue, purple, and green vertical lines indicate CH_4_ + CO_2_ injection, H_2_ injection, sulfate total consumption, and formation water reinjection, respectively.

The quantities of benzene and toluene measured followed the same trends. Benzene and toluene levels in the gases slightly decreased over the first 21 days (−7∙10^−3^ ± 4∙10^−3^ mmol between day 3 and day 21 for both benzene and toluene; Figure 2). Afterward, the concentrations obtained were held constant, considering the standard deviations. In the liquid phase, the quantities measured were constant throughout the experiment, considering the standard deviations. A closer look at the behavior of benzene and toluene in the aqueous phase shows that the carbon isotope values for benzene and toluene did not vary within the standard deviations (Table 2). From day 21 onward, the carbon isotope ratios of benzene and toluene slightly changed compared with those determined between day 3 and day 15, when benzene and toluene were enriched by gaseous inputs.

**Table 2:** Isotopic ratios of benzene and toluene during the experiment.

Day of experiment	δ^13^C/^12^C Isotopic ratio (‰)			
	Liquid phase		Gas phase	
	Benzene	Toluene	Benzene	Toluene
3	−28.4 ± 0.3	−27.25 ± 0.05	−25.9 ± 0.3	−25.94 ± 0.09
8	−28.0 ± 0.6	−27.1 ± 0.1	−26.6 ± 0.4	−25.0 ± 0.2
11	−27.86 ± 0.07	−27.14 ± 0.06	−25.1 ± 0.9	−25.6 ± 0.7
15	−28.0 ± 0.1	−27.2 ± 0.1	−26.1 ± 0.6	−25.88 ± 0.02
21	−27.80 ± 0.06	−27.08 ± 0.09	−26.5 ± 0.5	−25.0 ± 0.2
73	−27.6 ± 0.2	−26.6 ± 0.2	−25.6 ± 0.2	−26.3 ± 0.2
102	−27.5 ± 0.2	−26.5 ± 0.1	−25.5 ± 0.8	−25.3 ± 0.4

### 3.4 Microbial community evolution during the experiment

**Figure 3:**
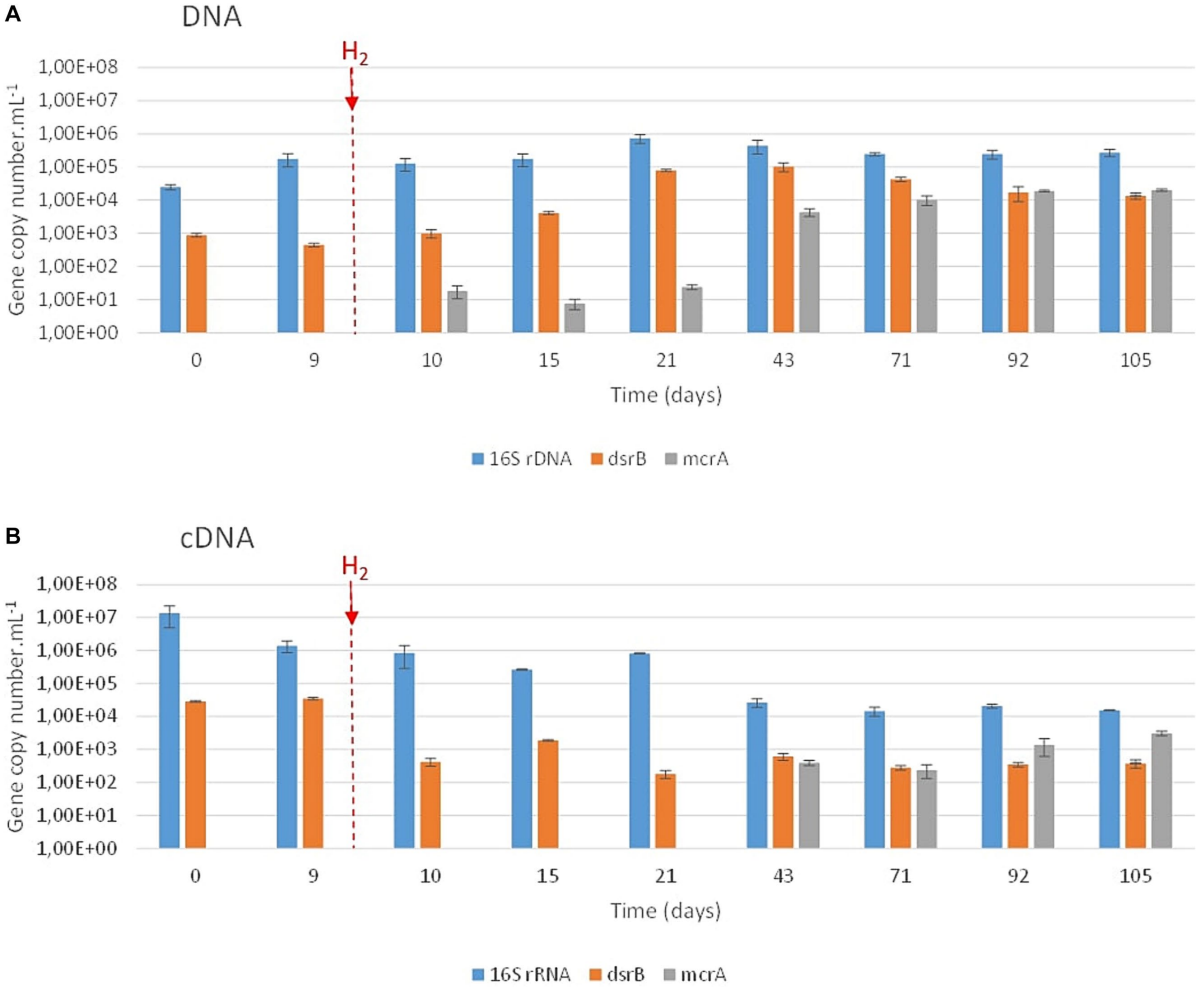
qPCR quantification of prokaryotes, sulfate reducers and methanogens over incubation time. The various microbial groups were targeted via the *16S rRNA* (prokaryotes; in blue), *dsrB* (sulfate reducers; in orange) and *mcrA* (methanogens; in gray) genes **(A)** and their transcripts **(B)**. H_2_ injection is indicated by the vertical red hatched line.

During the experiment, the prokaryotic concentration increased from 2.4∙10^4^ ± 4.3∙10^3^ copies of *16S rRNA* genes.mL^−1^ at the beginning of the experiment to a maximum of 7.4∙10^5^ ± 2.2∙10^5^ copies of *16S rRNA* genes.mL^−1^ after 21 days of incubation (Figure 3A). After 105 days of incubation, the average concentration of *16S rRNA* genes was 2.7∙10^5^ ± 8.8∙10^4^ copies.mL^−1^. Based on the results of the *dsrB* gene quantification, sulfate reducers were present throughout the experiment despite the total sulfate consumption after day 20. A complementary experiment using *Desulforamulus profundi* Bs107 as a model sulfate reducer demonstrated *dsrB* gene expression even in the absence of sulfate (data not shown). After H_2_ injection on day 9, the concentration of sulfate-reducers continuously increased from 4.4∙10^2^ ± 6.0∙10^1^ (day 9) to 1.0∙10^5^ ± 3.1∙10^4^ copies of the *dsrB* genes.mL^−1^ (day 43). Based on the *mcrA* gene, methanogens began to be detected on day 10 (1 day after H_2_ injection), with 1.8∙10^1^ ± 7.7∙10^0^ copies of the *mcrA* gene.mL^−1^. The concentrations of these archaea remained quite low until day 21 (<2.4 10^1^ copies of the *mcrA* gene.mL^−1^). Then, they reached a maximum on day 71, with 1.0∙10^4^ ± 3.3∙10^3^ copies of the *mcrA* gene.mL^−1^. Their concentrations remained stable until the end of the experiment. Microbial activity decreased throughout the incubation period (Figure 3B), as did the sulfate-reducing activity. From day 43 onward, methanogens showed increased activity within the microbial community.

**Figure 4:**
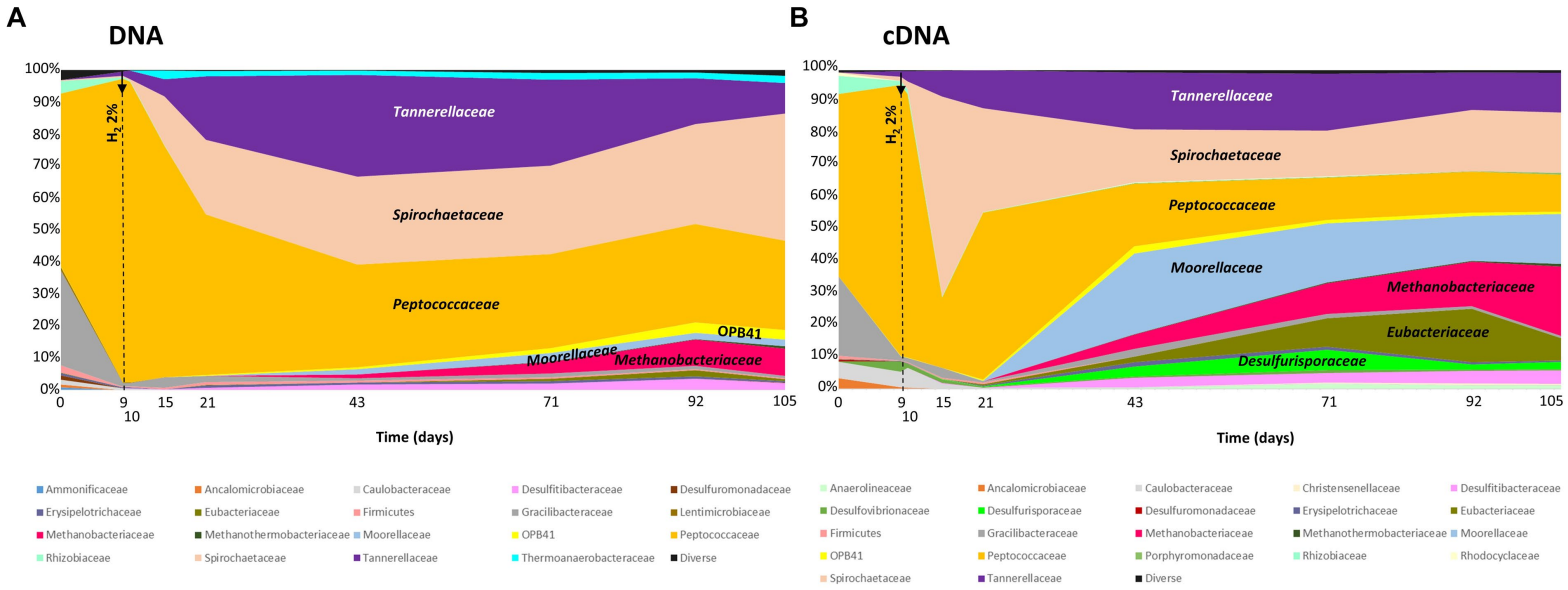
Monitoring the taxonomic diversity of microorganisms evolving in the high-pressure reactor during simulated H_2_ injection in a deep aquifer used as a UGS. **(A)** Analysis of prokaryotic diversity based on the *16S rRNA* gene; **(B)** Analysis of prokaryotic diversity based on transcripts of the same gene. The results are expressed as relative proportions. The vertical hatched line indicates the day of H_2_ injection. The names of the major families are indicated in the figures.

The taxonomic diversity of the microbial community was monitored during the experiment by high-throughput sequencing of the *16S rRNA* gene (Figure 4). Initially, the microbial community was dominated by the *Peptococcaceae* (54% relative representativeness), *Gracilibacteraceae* (30%), *Rhizobiaceae* (4%), and *Tannerallaceae* (2%) families (Figure 4A). Just before H_2_ injection (day 9) and 1 day after, the largely dominant family was *Peptococcaceae*, with 95% and 94% relative representativeness, respectively. After 21 days of incubation, i.e., 12 days after H_2_ injection, the microbial community was dominated by the *Peptococcaceae* (50%), *Spirochaetaceae* (fermentative bacteria; 23%) and *Tannerellaceae* (20%) families. Among the *Spirochaetaceae*, the most abundant ASV could be affiliated with *Rectinema cohabitans* HM (100% identity on 366 nt). By day 43, members of three other families began to grow until day 105: *Anaerosomataceae* (OPB41; 3%), *Moorellaceae* (2%) and *Methanobacteriaceae* (8%). The *Peptococcaceae* family was almost exclusively represented by sulfate-reducing bacteria close to the *Desulforamulus reducens* species until day 10. From day 15, the detection of formate led us to analyze the microbial diversity to identify the producer of this molecule. At this time, bacteria affiliated with a second taxonomic group of *Peptococcaceae* began to grow. This group is phylogenetically close to a strain previously isolated from the same aquifer, Ab_L_15_s1 (377 nt; 100% identity), which has not been described and whose closest genus is *Phosphitispora* (1,348 nt; 94%; personal communication). As incubation progresses, these bacteria became dominant within *Peptocococcaceae*, representing 44 to 81% of the sequences affiliated with this family. Finally, a third group of *Peptococcaceae*, close to the sulfate-reducing species *Desulforamulus profundi* Bs107 (377 nt; 100%), developed from day 21 and represented 9 to 13% of the representatives of this family until the end of incubation. Regarding active microorganisms (Figure 4B), in addition to the described families, there were two other families active after H_2_ injection: *Tannerellaceae* (fermentative bacteria), *Spirochaetaceae* (fermentative bacteria), *Peptococcaceae* (sulafe reducers and fermenters)*, Moorellaceae* (acetogens), *Eubacteriaceae* (fermentative bacteria) and *Desulfurisporaceae* (sulfate reducers). Throughout the experiment, the microbial community was largely dominated by bacteria, and methanogenic archaea accounted for only 8% of the relative representativeness at the end of incubation. Most members of the *Methanobacteriaceae* family are affiliated with the species *Methanobacterium flexile* (381 nt; 100%; accession #NR_116276).

### 3.5 Solid phase evolution during the experiment

**Figure 5:**
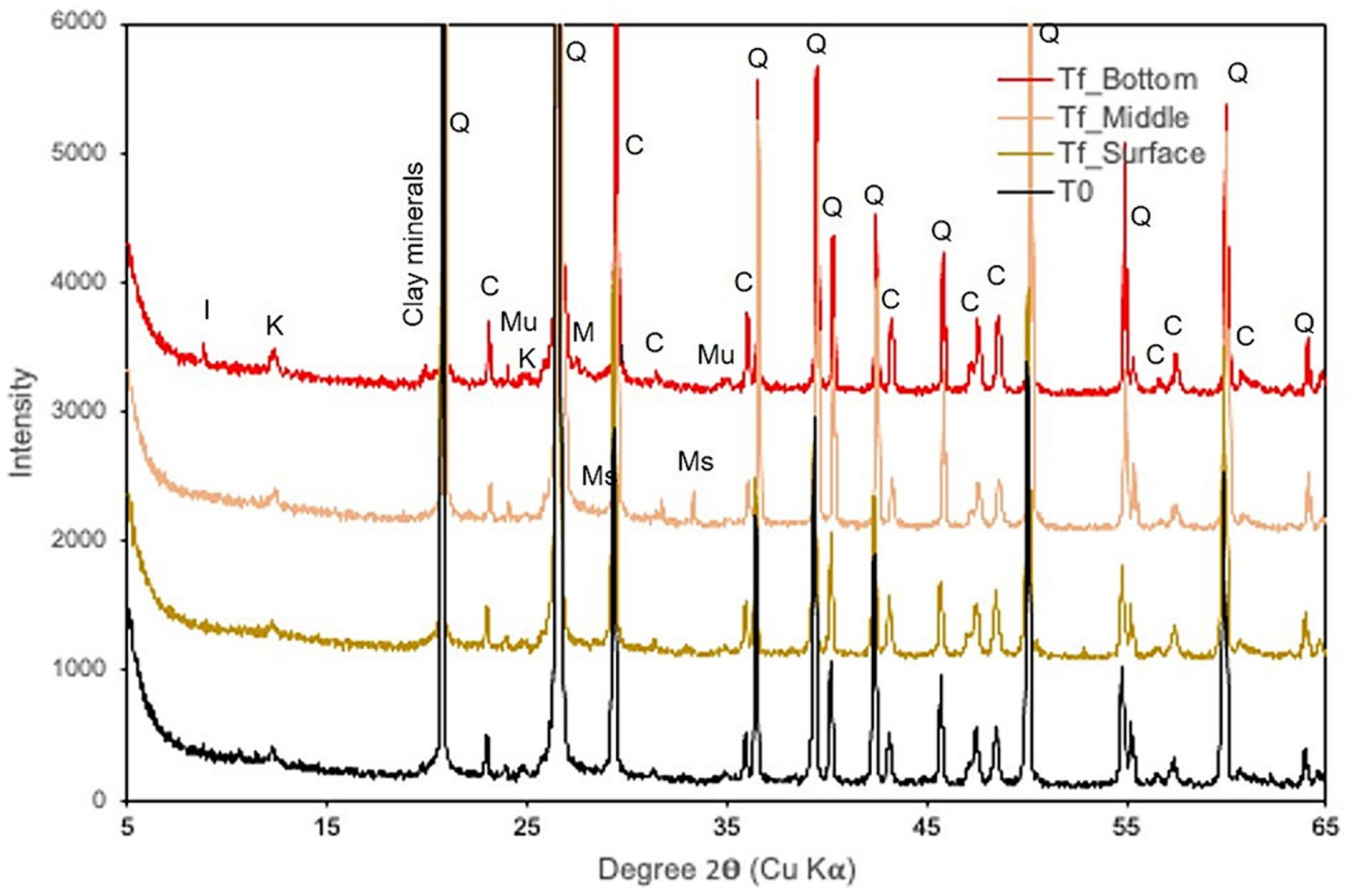
X-ray diffraction patterns of samples collected at the beginning (T_0_) and at the end of the experiment (T_f_) at various reactor depths. I, illite; K, kaolinite; Q, quartz; C, calcite; Mu, muscovite; Ms., Marcasite.

XRD revealed quartz in the initial rock (80%) with calcite (12%), muscovite (4%), and clay minerals, including illite and kaolinite, as well as traces of iron sulfite (marcasite; Figure 5). At the end of the experiment, the same phases were detected. Note that illite clay minerals seemed to increase slightly at the bottom of the basket, while iron sulfides increased in the middle.

**Figure 6:**
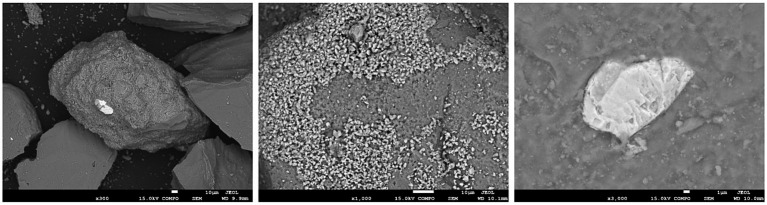
SEM image of variably coated quartz grains. Note the presence of an iron sulfide grain in white (left). Close-up of micrite grains forming a quartz grain coating, with molds of former micrite grains on the quartz surface (middle). Close-up of an iron sulfide grain of detrital origin partially embedded in quartz (right).

SEM–EDS observations showed that the solid phase was mainly composed of quartz (Figure 6). The grains were variably coated with micritic calcite and, to a lesser extent, clays, with both phases commonly mixed. The molding of former micrite grains on the surface of several quartz grains indicated an episode of quartz overgrowth after micrite emplacement. Other minerals presented in trace amounts included iron sulfides and, more rarely, K-rich silicates (identified as muscovite by XRD), barite, rutile, and iron oxides. All these phases were observed as parts of the coatings. Although the well-preserved euhedral morphology of the micrites might have suggested authigenic growth, most of the iron sulfide grains were interpreted as being of detrital origin. No marked changes could be observed before or after the experiment.

### 3.6 Biochemical modeling

**Figure 7:**
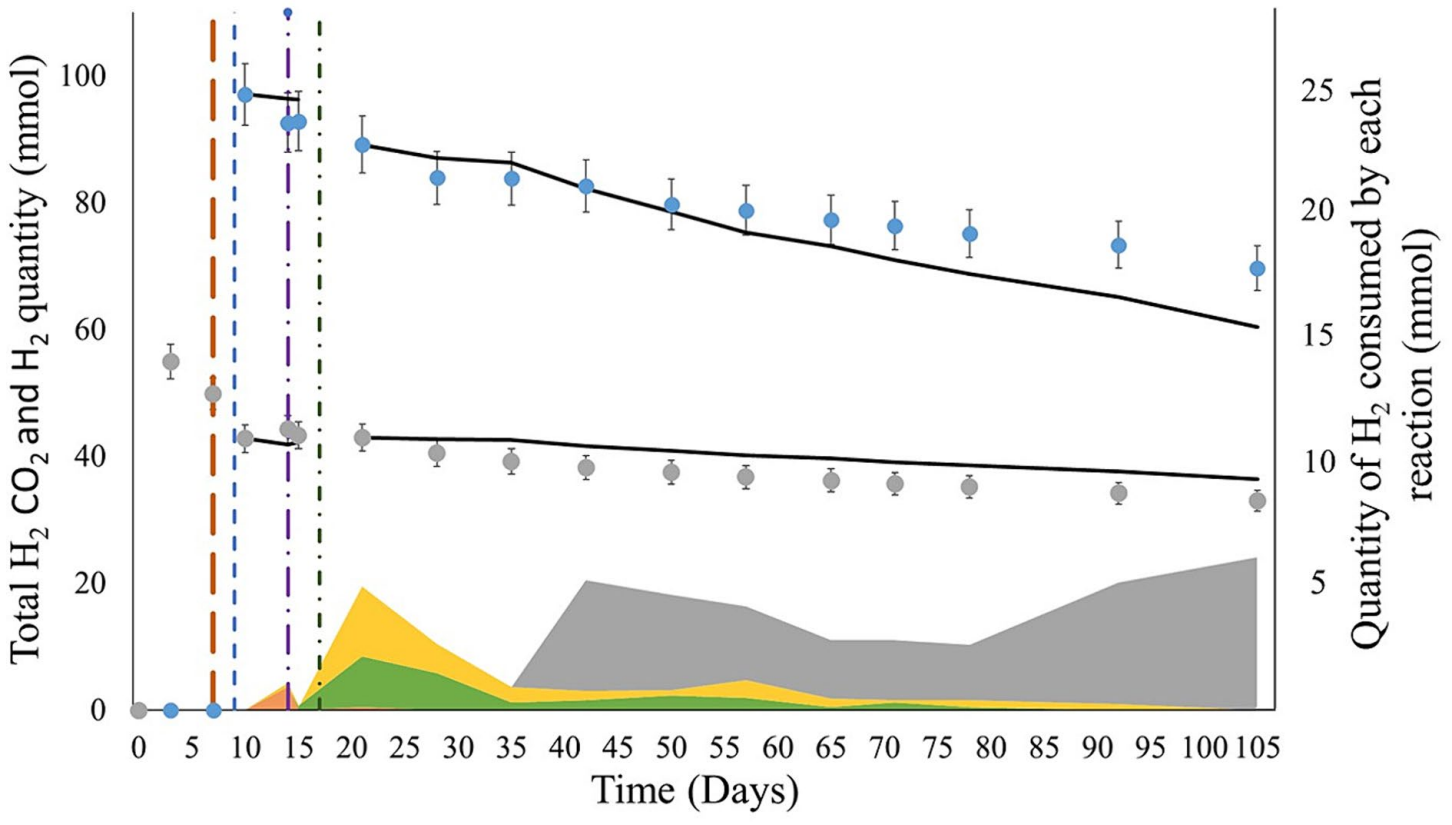
Comparison of the evolution of the experimental and modeled H_2_ and CO_2_ quantities during the experiments. The blue and gray dots represent the evolution of the total experimental quantities of H_2_ and CO_2_ (liquid + gas phase), respectively. The black curves show the quantities of H_2_ and CO_2_ modeled by calculating the extent of the reaction. The brown, blue, purple, and green vertical lines indicate the CH_4_ + CO_2_ gas mix reinjection, H_2_ injection, total sulfate consumption, and formation water reinjection, respectively. The orange, yellow, green, and gray areas correspond to the H_2_ quantities consumed by sulfate reduction, acetogenesis, formate production, and methanogenesis, respectively, between each measuring point.

To calculate the extent of each metabolic reaction, the reactions cited in section 2.2.6 were considered during the periods when the microorganisms used were detected. Sulfate reduction, acetogenesis, formate production, and methanogenesis were considered from days 9 to 15, 9 to 105, 9 to 105, and 35 to 105, respectively. From H_2_ injection on day 9 to total sulfate consumption, H_2_ was consumed only by sulfate reducers (3.5∙10^−3^ ± 1.8∙10^−4^ mol; Figure 7). From day 15, H_2_ was consumed through acetogenesis (5.4∙10^−3^ ± 2.7∙10^−4^ mol) and formate production (4.5∙10^−3^ ± 2.2∙10^−4^ mol). From day 35 to the end of the experiment, methanogenesis explained most of the H_2_ consumption (2.5∙10^−2^ ± 5.0∙10^−3^ mol). Overall, methanogenesis was the primary source of H_2_ consumption. While H_2_S was not detected in the gas phase, based on sulfate reduction after day 9, 8.8∙10^−4^ ± 4.4∙10^−4^ mol of sulfide should have been produced.

### 3.7 *In situ* characterization protocol

**Figure 8:**
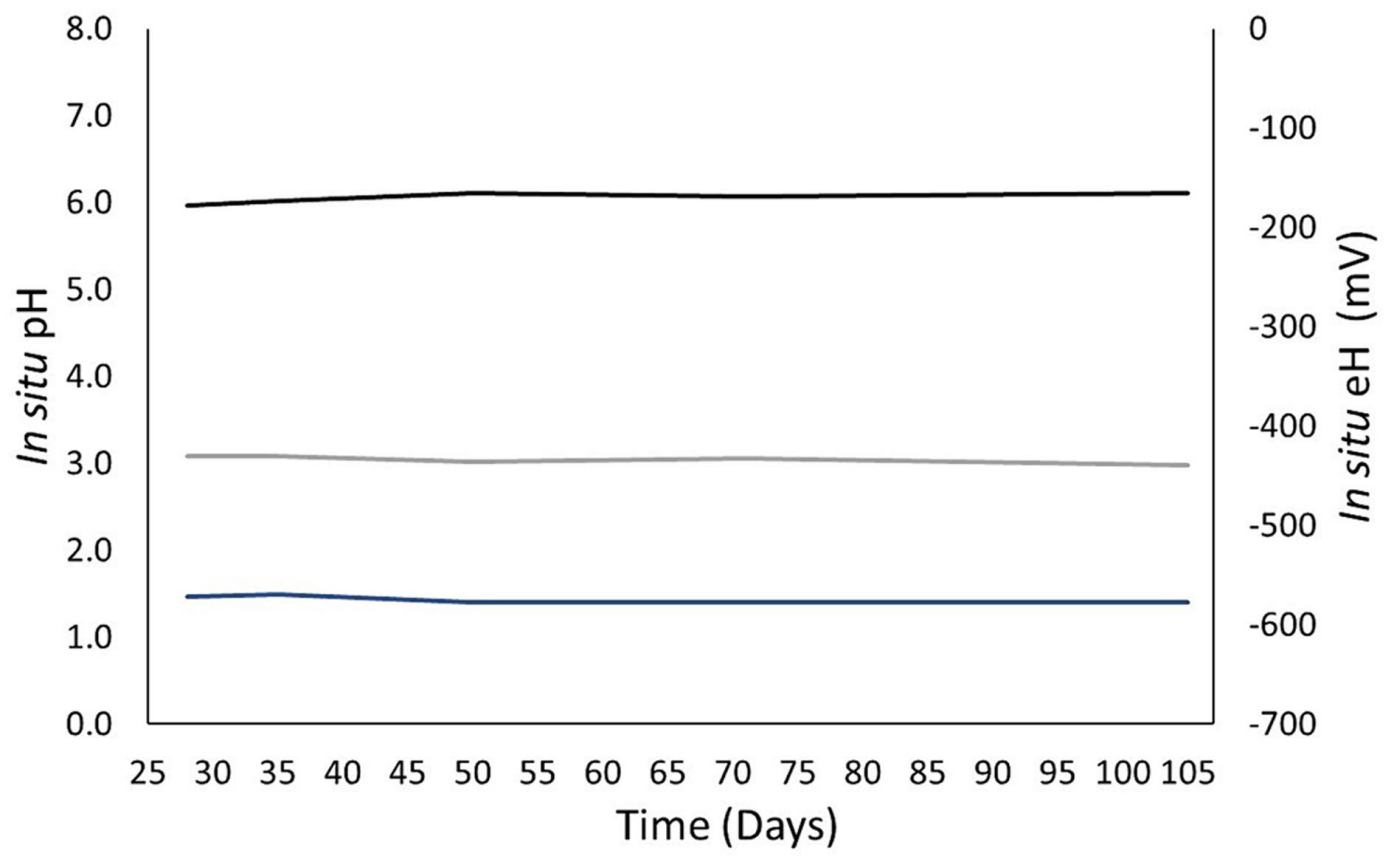
Evolution of the *in situ* pH and oxidoreduction potential computed with the *in situ* characterization protocol developed in this work. The black curve represents the calculated pH. The gray and blue curves correspond to the carbon and hydrogen redox potentials calculated by the Nernst equation.

On the first sampling with this protocol, on day 28, the pH obtained was 6.0 (Figure 8). This value did not significantly evolve throughout the experiment, as the evolution of the solution composition was slow. All the pH values were between 6.0 and 6.1. The values measured at atmospheric pressure were between 6.6 and 7.2. Gas solubilization caused acidification of the solution. The carbon and hydrogen Nernst potentials were distinct, directly showing the lack of equilibrium among the redox couples. For pH, the values were stable for each potential. The carbon and hydrogen ranged from −439 to −430 mV and −577 and −572 mV, respectively. The uncertainty of these values was calculated with the combined uncertainties of the measures used in this protocol. The uncertainty of the pH was ±0.1 and was mainly due to the uncertainty in the quantity of CO_2_ and bicarbonate. The uncertainty of the Nernst potential was ±10 mV, which was caused mainly by the uncertainty of the pH.

## 4 Discussion

The H_2_ geological storage simulated in this experiment has the particularity of targeting a low-salt aquifer (Ab_L_1) with sulfate concentrations below 7 mg. L^−1^ (Haddad et al., 2022a, 2023). At the beginning of incubation, the gas phase consisted of 99% CH_4_, 1% CO_2_, 7.95 ppm benzene, and 3.57 ppm toluene at a pressure of 60 bar and a temperature of 36°C. Some of the CO_2_ rapidly solubilized in water. Storage of the aquifer formation water containing the microorganisms most certainly resulted in an evolution in its taxonomic composition, although the sample was kept at 4°C to slow metabolism.

### 4.1 Sulfate and acetate evolution

In the formation water, sulfate was the main external electron acceptor available to microorganisms and was most certainly depleted, explaining why, at the beginning of the experiment, the microbial community was largely dominated by bacteria capable of fermenting the organic molecules present or necromass. Among these microorganisms, the amplicon sequence variants (ASVs) grouped in the *Peptococcaceae* family were, for the most part, affiliated with the sulfate-reducing *Desulforamulus reducens* species, described as being capable of fermenting simple compounds, such as pyruvate (Visser et al., 2016). At the beginning of incubation, the microbial community returned to conditions close to those *in situ* (temperature, pressure, rock, etc.) with a gas phase simulating natural gas storage. *D. reducens* was the main sulfate reducer present, completely consuming 0.99 mmol of sulfate in just 15 days. This sulfate reducer likely operated mainly heterotrophically prior to H_2_ injection. This species has been shown to incompletely oxidize organic molecules serving as carbon sources (Visser et al., 2016) and could therefore be one of the sources of acetate. Furthermore, the measured sulfate consumption (0.6 mmol) and acetate production (1.2 mmol) between days 3 and 10 are consistent with this metabolic stoichiometry (Daumas et al., 1993). This metabolic activity could also partly explain bicarbonate production in the incubation phase. Members of the *Gracillibacteriaceae* and *Spirochaetaceae* (i.e.*, Rectinema cohabitans*) families also participate in acetate production via fermentation (Lee et al., 2006; Haddad et al., 2022b). As soon as sulfate was depleted, the relative abundance of *D. ramulus* species declined sharply from almost 100% of the relative abundance of *Peptococcaceae* to 7%.

### 4.2 Formate evolution

Formate began to be detected on day 15, i.e., 6 days after H_2_ injection. Previous studies have shown that formate appears very quickly after H_2_ addition under conditions similar to those in our experiment (Haddad et al., 2022b; Mura et al., 2024). In this study, however, sulfate was depleted by day 15. Based on the work of Visser and collaborators (2016), we hypothesize that *D. reducens* can use formate produced between days 9 and 15 as a carbon source as long as sulfate is present as an electron acceptor. This would explain the apparent delay in formate production in this experiment and the sharp increase between days 15 and 21. For the formate source, *Peptococcaceae*, close to strain Ab_L_15_s1, developed from day 15 and remained active until the end of incubation. As hypothesized, pressure incubation with CO_2_ is thought to promote formate production during acetogenesis (Haddad et al., 2022b; Mura et al., 2024). The *Peptococcaceae* strain Ab_L_15_s1 could therefore be a good candidate in the search for the microorganism responsible for formate production in this study. The low equilibrium constant for formate production (Stams, 1994) could explain why formate was not observed in near atmospheric pressure experiments. Formate production at high pressure could be triggered by equilibrium displacement due to high H_2_ partial pressure. Under the conditions of this study, the calculated equilibrium molality of formate enabled by thermodynamic equilibrium just after H_2_ injection (day 14) was 6.7 mmol.kg^−1^, whereas this value would be negligible for the same H_2_ molar fraction at atmospheric pressure (0.1 mmol.kg^−1^). This could explain why formate is not detected in near atmospheric pressure experiments. The decrease in the formate production rate observed at the end of the experiment could be caused by thermodynamic constraints as the reaction approaches equilibrium (Jin and Bethke, 2005). Finally, the species *Methanobacterium flexile*, family *Methanobacteriaceae*, has been described to utilize H_2_/CO_2_ or formate (Zhu et al., 2011). Moreover, methanogenesis is an alkalinity-producing metabolic process. It was previously described that H_2_ or formate utilization in *Methanococcus thermolithotrophicus* was pH dependent, with formate utilization favored at higher pH (Belay et al., 1986). However, pH monitoring in the bulk appeared to be constant throughout the experiment (pH 6.0–6.1). Different pH values cannot be ruled out in microniches with different rock porosities, but this cannot be verified. Another hypothesis could be that formate consumers need time to adapt. Notably, the members of the OPB41 taxon (family *Anaerosomataceae*), detected from day 43 onward, have been shown to grow lithotrophically on H_2_ or formate (Khomyakova et al., 2022).

### 4.3 Evolution of CO_2_, H_2_, CH_4_, and H_2_S

The physicochemical monitoring of CO_2_, H_2_, CH_4_ and H_2_S throughout the experiment revealed no substantial changes. Although it was not possible to measure the production of CH_4_ and H_2_S, it is nevertheless certain that these molecules were produced by microbial metabolism. Fermentation products include CO_2_, H_2_, and organic acids such as acetate. Furthermore, the modeled H_2_ consumption based on sulfate-reduction, methanogenesis, acetogenesis and formate production extents of reaction was overestimated, which could support the fact that fermenters produced H_2_. Methanogens produced methane, as the calculated extent of acetogenesis was insufficient to explain the global CO_2_ consumption. As for methanogens, they obviously produced methane, as the calculated acetogenesis extent of reaction was insufficient to explain the global CO_2_ consumption. Extents of reaction calculations suggest that methanogenesis was responsible for the major part of H_2_ consumption. As in a study (Haddad et al., 2022b) with a similar sulfate concentration before H_2_ injection (1.2 mM), H_2_S was not detected in the gas phase. The low but continuous release of iron during the experiment and the detection of a potentially greater amount of marcasite at the end of the experiment suggest the attenuation of sulfide by iron sulfide precipitation. We hypothesize that this release could occur by partial dissolution of iron-bearing minerals, possibly aluminosilicates and/or clays.

### 4.4 Calcite dissolution

Calcium and bicarbonate release in the aqueous phase occurred only during the incubation phase, implying that calcite dissolution was triggered by the equilibration of the formation water with the rock. Indeed, the calculated *in situ* pH was two units lower than the measured pH of the initial water mix at atmospheric pressure, mainly because of CO_2_ degassing during the depressurization of the bottom hole samplers. We hypothesize that calcite precipitated during formation water sampling and dissolved after the initial CO_2_ injection. Thus, this phenomenon was not linked to H_2_ injection and should not lead to porosity changes in the reservoir. Globally, changes in mineralogy remain small and do not indicate a risk of variation in the petrological properties during storage.

### 4.5 Benzene and toluene evolution

Benzene and toluene were added to the gas phase to obtain conditions similar to those in the field (Aüllo et al., 2016). These monoaromatic compounds are also present in water due to thermodynamic equilibrium. These compounds were monitored in both the liquid and gas phases. The variations observed during the first few days could be caused by adsorption on the solid surface and the liquid–vapor equilibrium. The isotopic values of carbon fluctuate around −27.9 ± 0.3 ‰ and – 27.0 ± 0.3 ‰ for benzene and toluene, respectively, in water, which does not indicate enrichment or depletion. The conditions of the experiment (duration and addition of water and gas containing benzene and toluene) impact the evidence of biodegradation, as the concentration of benzene and toluene in the water depends on the thermodynamic equilibrium with the gas phase (Darracq et al., 2009). The experiment did not last long enough to demonstrate biodegradation, as benzene degradation by sulfate-reducing bacteria is slow compared to other metabolic processes (Mancini et al., 2003).

### 4.6 Fate of a UGS coinjected with 2% H_2_

In this study, the scenario tested was the coinjection of 2% H_2_ with a gas mixture simulating natural gas (99% CH_4_, 1% CO_2_, traces of BT). Without an anomaly, the storage site we experimentally simulated is in a low-salt water and, therefore, has a very low sulfate concentration. Deep aquifers are oligotrophic, and the presence of natural gas in the vicinity has been shown to increase microbial activity, particularly sulfate reduction, due to the dissolved organic molecules of the gas in the formation water, even if some of these molecules are recalcitrant to biodegradation (Ranchou-Peyruse et al., 2019). Thus, even the sulfate renewed by the slow recharge of water could not compensate for the decrease in its concentration over the years. During massive H_2_ injection, it is therefore expected that the only two electron acceptors will be sulfate and CO_2_. In view of the results presented here, it seems likely that sulfate will be depleted very quickly since there are few sulfated minerals (Figure 1). A nonnegligible proportion of the sulfide will remain trapped in the rock as iron sulfide (Figure 6). This means that the last available electron acceptor remaining, and therefore a limiting growth factor, will be CO_2_. Dihydrogen and CO_2_ are then consumed by acetogenic and methanogenic microorganisms. In our case, this consumption appeared to be limited over the incubation period of approximately 3 months. There are other limiting nutrients that slow microbial development and seem to prevent it from exceeding a maximum concentration of microorganisms (Figure 3). The low metabolic efficiency of sulfate reduction, acetogenesis, and methanogenesis observed here contrasts with results for other UGSs simulated in high-pressure reactors and showing alkalinization phenomena (Haddad et al., 2023; Mura et al., 2024).

## 5 Conclusion

In this study, the injection of a natural gas/H_2_ blend (98% / 2%) in a low-salinity aquifer was simulated in a high-pressure reactor. This work provides more data for understanding H_2_ behavior in deep aquifers and helps researchers understand the parameters affecting site variations. Overall, this experiment suggested that H_2_ coinjection with natural gas in this UGS could be viable due to several factors:

- Contrary to experiments using the same protocol, while sulfate reducers and methanogens were active, only a minor amount of H_2_ was consumed over the 3 months of the experiment. This probably occurred due to a lack of nutrients.
- Dissolution and precipitation of minerals likely occurred but were not formally detected. As for other experiments, the impact of H_2_ on the aquifer porous rock is negligible, as no significant changes were observed in the XRD and SEM analyses.
- After initial CO_2_ solubilization from the injected CH_4_/CO_2_/benzene/toluene gas mixture, no significant changes in the reactor *in situ* pH or redox potential were observed due to low microbial activity. Thus, calcium or magnesium carbonate precipitation was prevented.

Although we identified key parameters for viable H_2_ costorage in deep aquifers, quantitative extrapolation of these results to the reservoir remains a challenge due to scale effects. Notably, the conditions simulated in the reactor represent those encountered near the gas bubble, but due to transport phenomena, the conditions far from the gas bubble could be very different. Thus, the modeling of pilot-scale studies will be essential for accurate feed reservoir simulations of H_2_ costorage in aquifers.

## Data availability statement

The datasets presented in this study can be found in online repositories. The names of the repository/repositories and accession number(s) can be found at: https://www.ncbi.nlm.nih.gov/, PRJNA1117242.

## Author contributions

JM: Writing – review & editing, Writing – original draft, Visualization, Software, Methodology, Investigation, Formal analysis, Data curation. MR-P: Writing – review & editing, Writing – original draft, Visualization, Validation, Supervision, Methodology, Investigation, Formal analysis, Data curation, Conceptualization. MG: Writing – original draft, Methodology, Investigation, Formal analysis, Data curation. MD: Writing – original draft, Supervision, Methodology, Data curation. ML: Writing – original draft, Methodology, Investigation, Data curation. M-PI: Writing – review & editing, Writing – original draft, Visualization, Validation, Methodology, Investigation, Data curation. IH: Writing – review & editing, Writing – original draft, Validation, Methodology, Investigation, Data curation. GH: Writing – review & editing, Writing – original draft, Validation, Methodology, Investigation, Data curation. MP: Writing – original draft, Methodology, Investigation, Data curation. MdSB: Writing – original draft, Methodology, Investigation, Data curation. PCh: Writing – original draft, Validation, Resources, Funding acquisition, Conceptualization. GC: Writing – original draft, Validation, Resources, Funding acquisition, Conceptualization. AP: Writing – original draft, Validation, Resources, Investigation, Funding acquisition, Conceptualization. PCe: Writing – review & editing, Writing – original draft, Validation, Supervision, Resources, Project administration, Methodology, Investigation, Funding acquisition, Conceptualization. AR-P: Writing – review & editing, Writing – original draft, Visualization, Validation, Supervision, Resources, Project administration, Methodology, Investigation, Funding acquisition, Formal analysis, Conceptualization.
